# The Ups and Downs of a Medical Referee

**Published:** 1990-09

**Authors:** James C Briggs

**Affiliations:** Consultant Histopathologist, Frenchay Hospital, Bristol BS16 1LE


### The Ups and Downs of a Medical Referee

**James C Briggs, MB, BS, DPath, FRCPath**
Consultant Histopathologist, Frenchay Hospital, Bristol BS16 1LE

Cremation of the dead has been legal in Great Britain only since the end of the last century, when the activities of an eccentric General Practitioner in South Wales precipitated a crisis. According to Bernard Knight (1987), "this eccentric family doctor, towards the end of a colourful career, became the father of a small son named Jesus Christ Price. When the infant died, the father publically cremated him in a field in full view of a departing chapel congregation. The public outrage caused him to be arrested, but he conducted his own defence at Cardiff Assizes and was acquitted. This became a precedent for cremation and the first Cremation Act was passed in 1902." As part of this Act, the appointment of Medical Referees to crematoria was established. The Medical Referee is a doctor attached to the cremation authority and who must have not less than five years' standing and relevant experience and qualifications. (BMA, 1988).

I have been one of the Medical Referees for the City of Bristol for the past six years or so. Bristol has a Chief Medical Referee and two Assistants; I am one of the latter. Medical Referees are appointed by the Crown, through the Lord Chancellor's Office with the responsibility of vetting all the applications for cremation which arise within their area of jurisdiction. The job appears to be for life, or until given up by mutual agreement! The work is shared between the three of us in Bristol and I am responsible for the overseeing of the paper work on two days a week. In practice I am hardly ever disturbed during the working day; the relevant sets of papers are delivered to me from the Crematorium by about 6.00 pm on the appropriate days and I have to satisfy myself that everything is in order, or have sorted out the problems, by early next morning when the papers have to be back in the Crematorium.

The paper work is varied but basically consists of what are technically known as forms A, B, C and F. These are accompanied by a Certification of Death, but not the Death Certificate, issued either by the Registrar of Births and Deaths, on a green piece of paper, or by the Coroner, on a yellow piece of paper (officially this is Form E). Without a Certificate of Death none of the other paper work is valid. The Medical Referee never sees the actual Death Certificate, as filled in by a Medical practitioner in non Coroner's cases. He also hardly ever knows the cause of death in Coroner's cases which had post mortems. The only information is on the paper work supplied by the Crematorium.

Form A is the application for the cremation of the deceased. This application is usually by a member of the family, or an executor. Occasionally it is filled in by a Local Government or Hospital official if no next of kin can be found. The applicants have to answer ten questions and their signature has to be witnessed by an appropriate authority, usually the Undertaker arranging the funeral.

Forms B and C are colloquially known to most doctors as Parts One and Two of the cremation certificate. Form B consists of twenty questions, some subdivided, to be answered by a medical practitioner who has been looking after the deceased, but not necessarily the person who has issued the death certificate. The doctor signing form B is required to print his name and give his address and telephone number, as well as his signature. Rather stupidly, the City of Bristol version of the form has yet another question after the doctor has signed that he has completed all the answers! This question involves the presence of pacemakers or other types of implant and is placed almost at the bottom of the second page of the Form, underneath the doctor's signature. Where patients die in hospital, form B is usually filled out by one of the junior doctors and this battery of questions is a source of numerous potential and real mistakes for the uninitiated! Form A is also somewhat daunting, especially as the Applicant for cremation has almost never filled in such a form before; however form C, the second of the medical certificates is much less open to incorrect answers. Firstly, the number of questions is relatively small, only eight and, secondly, the form is only allowed to be signed by a medical practitioner of at least five years full registration who, by this stage, has usually learnt some of the ropes! Again the doctor signing the certificate is asked to print his name and supply an address with a telephone number. On this occasion there are no further questions under the signature!

Form F is the one on the back of the two cremation certificates B & C. This form is reserved for the Medical Referee and is signed when the Referee is happy that all the questions on forms A, B and C have been correctly answered. These forms amount to a total of about forty questions to be checked in each case, but forms B & C are not needed in Coroner's cases which had autopsies.

The above protocol of paper work is the commonest, but not the only type presented to the Medical Referee. Special forms are used for, among other things, still-births, people dying abroad, nearly always holidaymakers, and for the final disposal of the remains of those people who have donated their bodies for dissection by medical students under the Anatomy Act.

When I became Medical referee, at the end of March 1984, I decided to keep a simple record of the number of cases that I had dealt with and the number of queries raised by those cases. Some of these queries require a telephone call which, by virtue of the timetable placed upon the Medical Referee, have to be made in the evenings. One of the great problems with people dying in hospital is that the signatories of forms B and C often give only their hospital telephone number. Frequently they are off-duty in the evening and the hospital switchboard has no record of their private numbers. This leads to regular difficulties in sorting out problems on forms B & C. Any problem on form A is almost always insoluble in the evening, since it is filled in by an Applicant who may or may not be a member of the family and usually witnessed by the Undertaker; no telephone numbers are given and the name of the Undertaking firm is not asked for! In these instances, and some others, the Crematorium has to sort things the next morning before cremation can proceed. Although I kept records of total case and problem numbers, it was only in the year 1986–87 that I noted details of the problem types.

In 1984–85 I dealt with a total of 1,280 sets of papers on 90 days of the year, a rate of 14.2 sets per day. There were 36 problems which needed clarification.

In 1985–86 I covered 85 days and 1,383 applications, ie 16.3 per day. 44 problems occurred.

There were 84 working days in 1986–87 with 1,325 applications (15.8/day). The problem rate had risen to 144. I suspect this rise was mainly due to more meticulous record keeping on my part rather than an actual rise.

The equivalent figures for the years up to April 1990 are as follows.

1987–88. 1,468 applications on 88 days (16.7/day). 132 problems.
1988–89. 1,304 applications on 88 days (14.8/day). 134 problems.
1898–90. 1,419 applications on 87 days (16.3/day). 141 problems.

More than 75% of the problems are of an identical type and involve discrepancies in age on Forms A and B from the age given on the Certification of Death. I accept a difference of a year, since this can esily be due to lack of knowledge of the deceased's precise birthdate; others I have to clarify.

Between 3 and 5% of the difficulties are due to the doctor signing Form B not answering the "pacemaker" question. This always results in a 'phone call from me or the crematorium.

Two to 3% of "Cause of Death" answers are either unacceptable, or unclear. Occasionally there is a disparity between the answers on Forms B and C; these are usually resolvable with a 'phone call or two. In other instances the case is referred to the Coroner, eg as in one case where the doctors on both forms gave death as due to "1a. Bronchopneumonia. 1b. Fracture of femur." It is perhaps worth pointing out that this cause of death was accepted by the Registrar of Births and Deaths, otherwise the forms would not have come to me!!

Form A produces about 4% of the problems, but these, by virtue of the nature of the Form, are never due to medical insufficiency!

The question which causes most difficulty is one exclusively for the hospital doctor, and I see this mainly in my role as a Consultant Pathologist, rather than Medical Referee. Question 8A on Form B is a multi-part question, any part of which, when answered in the negative, causes the whole to become negative. The question itself concerns deaths of in-patients in Hospitals, the definition of a "hospital" being laid down (it is much wider than one might at first think); it goes on to enquire whether a post mortem has been held, and whether by a practitioner of five years full registration (who is not a relative of the deceased or a relative or partner of the doctor signing Form B) and whether the results of any post mortem are known to the doctor signing the Form. What is not clarified anywhere on either Form B or C is the implication of a "Yes" answer. In these circumstances there is no need for Form C! About 2.5% of the problems arise in this area. There would be more, but I manage to prevent almost any coming from my own hospital and presenting to the Referees.

The above problem is basically one of the design of the Forms (most of the questions seem to be unaltered from 1902!). Another design defect is that, certainly on City of Bristol Forms, there is no question along the lines of "If you have discussed the case with the coroner and he has allowed you to issue the Death Certificate then please so indicate." There are regular occasions when, from answers given on Form B, I know that the deceased died within a few hours of Hospital admission. Almost all Coroners wish to know of such cases, but in a significant percentage they will not order a post mortem and will allow the normal paper work to be completed (20% of Coroner's cases don't come to post mortem; Knight, 1987). Never-the-less the Coroner will record that he has been told of the case by completing a "Pink A" certificate. There is nothing on the Forms to prompt a doctor to record that he has discussed a case with the Coroner; in consequence I regularly inform the Coroner of these "sudden deaths", only to be told that he knows about all almost of them! However, there is at least one Crematorium Authority, in The Wirral, where this question is asked. Perhaps there are others.

Other regular but occasional problems arise; examples are:

a. Doctors forgetting to sign the Form.
b. Cause of Death not filled in.
c. Admitting to a pecuniary interest in the death (this absolutely rules out the signatory!!)
d. Doctors on holiday and not available to sign Form B when no other doctor had looked after the deceased (the signatory must have given some clinical care prior to death.)
e. Questions left unanswered.

All in all, the current design of the Forms required for Cremation leaves something to be desired. The task of the Medical Referee could be eased with better protocols and, as long as the existing Cremation Law remains, there will continue to be an important role for Referees. Whether the existing Law is suitable for today'3 / nother matter. In 1971 the Broderick Report suggested basic changes; to date virtually nothing has happened.

**References**

1. KNIGHT, BERNARD (1987) Legal Aspects of Medical Practice. 4th Edition. Churchill Livingstone.
2. Rights and Responsibilities of Doctors. (1988) BMA, London.

